# The quality of reporting of RCTs used within a postoperative pain management meta-analysis, using the CONSORT statement

**DOI:** 10.1186/1471-2253-12-13

**Published:** 2012-07-04

**Authors:** Victoria Borg Debono, Shiyuan Zhang, Chenglin Ye, James Paul, Aman Arya, Lindsay Hurlburt, Yamini Murthy, Lehana Thabane

**Affiliations:** 1 Department of Anesthesia, McMaster University, 2U1-1200 Main Street West, Hamilton, ON, L8N 3Z5, Canada; 2 Department of Clinical Epidemiology and Biostatistics, McMaster University, 1200 Main Street West, Hamilton, ON, L8N 3Z5, Canada; 3 Biostatistics Unit, Father Sean O'Sullivan Research Centre 3rd Floor Martha, Room H325, St. Joseph's Healthcare Hamilton, 50 Charlton Avenue East, Hamilton, ON, L8N 4A6, Canada; 4 Department of Medicine, Schulich School of Medicine, University of Western Ontario, 339 Windermere Road, London, ON, N6G 2 K3, Canada; 5 Department of Anesthesia, University of Toronto, Room 121, Fitzgerald Building, 150 College Street, Toronto, ON, M5S 3E2, Canada

## Abstract

**Background:**

Randomized controlled trials (RCTs) are routinely used in systematic reviews and meta-analyses that help inform healthcare and policy decision making. The proper reporting of RCTs is important because it acts as a proxy for health care providers and researchers to appraise the quality of the methodology, conduct and analysis of an RCT. The aims of this study are to analyse the overall quality of reporting in 23 RCTs that were used in a meta-analysis by assessing 3 key methodological items, and to determine factors associated with high quality of reporting. It is hypothesized that studies with larger sample sizes, that have funding reported, that are published in journals with a higher impact factor and that are in journals that have adopted or endorsed the CONSORT statement will be associated with better overall quality of reporting and reporting of key methodological items.

**Methods:**

We systematically reviewed RCTs used within an anesthesiology related post-operative pain management meta-analysis. We included all of the 23 RCTs used, all of which were parallel design that addressed the use of femoral nerve block in improving outcomes after total knee arthroplasty. Data abstraction was done independently by two reviewers. The two main outcomes were: 1) 15 point overall quality of reporting score (OQRS) based on the Consolidated Standards for Reporting Trials (CONSORT) and 2) 3 point key methodological item score (KMIS) based on allocation concealment, blinding and intention-to-treat analysis.

**Results:**

Twenty-three RCTs were included. The median OQRS was 9.0 (Interquartile Range = 3). A multivariable regression analysis did not show any significant association between OQRS or KMIS and our four predictor variables hypothesized to improve reporting. The direction and magnitude of our results when compared to similar studies suggest that the sample size and impact factor are associated with improved key methodological item reporting.

**Conclusions:**

The quality of reporting of RCTs used within an anesthesia related meta-analysis is poor to moderate. The information gained from this study should be used by journals to register the urgency for RCTs to be clear and transparent in reporting to help make literature accessible and comparable.

## Background

Transparent and well designed, conducted and reported randomised controlled trials (RCTs) are the gold standard in evaluating healthcare interventions [1]. The quality of reporting of RCTs in publications is important because it acts as a proxy for health care providers and researchers to appraise the quality of the methodology, conduct and analysis expected of an RCT [2-6]. The evaluation of the quality of reporting can be compromised when RCTs lack methodological rigour, thus generating biased results possibly resulting in exaggeration or shrinking of treatment effects [1,7-14]. Poor conduct in the quality of reporting of RCTs may allow the introduction of treatments that are less effective than the studies themselves report [13]. Hence, it is important to know the quality of RCTs used in meta-analyses in order to identify the reliability of the evidence that may inform treatment and care in medicine, including treatment in the realm of anesthesiology and pain management [4,7,12,15,16].

Readers and reviewers of published RCTs need complete, clearly written, and transparent information on a study’s methodology and findings in order to assess its quality [1]. Attempts in assessing the quality of reporting recurrently fail because authors of RCTs neglect to provide comprehensive and clear descriptions of critical methodological information [1,17-19]. In response to inadequate reporting the Consolidated Standards of Reporting Trials (CONSORT) group created the CONSORT statement and published its latest revision of the statement in 2010 [1,6]. The CONSORT 2010 Statement includes a 25 item checklist and a flow diagram. These are recommended items which should be incorporated into an RCT [1,6]. The statement provides guidance for reporting all RCTs with a focus on individually randomised, two groups, parallel trials [1].

Following the CONSORT initial statement publication in 1996, there has been improvement in the quality of reporting of some RCTs [1,10,20-22]. However, despite improvements noted, there continues to be observational studies reporting the inadequate quality of reporting of many RCTs [1,17], including RCTs in specialized journals [1,21,23-25]. There are a few other studies that have investigated the quality of reporting of RCTs specifically in anesthesia specialized journals. Many of them have evaluated the quality of reporting using items similar to items on the CONSORT 2010 checklist [7,26-28]. Some of the specialty journals in anesthesiology have also adopted or supported the CONSORT statement, yet the quality of reporting of parallel-group RCTs still remains poor [26]. Improvements made in the quality of reporting in the last decade may indicate a demand on the “quality, consistency and transparency” [26] of RCT reporting as well as endorsement of the CONSORT statement by medical journals which publish clinical trials [1,26]. The quality of reporting of RCTs may be improved in specialty journals pertaining to anesthesiology by clearly illustrating methodology. Areas in methodology requiring attention are allocation concealment (randomization blinding), blinding of data collectors and “discussion of Type II error in negative trials” [26].

We have two primary aims for this study. The first aim of this study is to analyse the overall quality of reporting and reporting of 3 key methodological items of 23 RCTs used in a meta-analysis that investigated analgesia outcomes in RCTs comparing femoral nerve block (FNB) with epidural and patient-controlled analgesia (PCA) after total knee arthroplasty [29]. Secondly, we aim to determine factors associated with higher quality of reporting. We hypothesized that studies with larger sample sizes, that have funding reported, that are published in journals with a higher impact factor and that are in journals that have adopted, endorsed or supported the CONSORT statement will be associated with better overall quality of reporting and reporting of the 3 key methodological items. Our study is unique in that we evaluated the quality of reporting of RCTs used within a meta-analysis using 15 items plus 3 methodologically related items, all of which were directly from the CONSORT 2010 checklist. It has been suggested that the quality of studies included in meta-analyses should be regularly evaluated as studies show that inadequate quality of trials may distort the results of systematic reviews and meta-analyses [9]. We consider it important to do an update on the status of the quality of reporting of RCTs published within anesthesia specialized journals as this will contribute more evidence around the quality of reporting of RCTs used. Much of our study’s method was modelled upon a previous study assessing the quality of reporting of RCTs in general endocrinology literature [3].

## Methods

### Study selection

The 23 RCTs included in this study for quality of reporting and key methodological item evaluation came from a meta-analysis that compared FNB, with and without sciatic nerve block, with epidural and PCA after total knee arthroplasty [29]. This meta-analysis included 23 RCTs for its own analysis. It was of interest to this study to know the reporting quality and reporting of key methodology items included in this meta-analysis. All 23 RCTs assessed were parallel-design and addressed the issue of comparative treatment effectiveness with respect to morphine consumption, incidence of side effects and pain experiences, which was scored on a pain scale both at rest and with activity [29].

### Data abstraction process

An electronic Excel standardized data collection form was used to extract data from each of the 23 RCTs. One pair of trained reviewers (VBD and SZ), both of whom were blinded to each other’s ratings, abstracted data independently. This form was pilot tested and subsequently modified for final revision. Any disagreement between the reviewers was rectified by consensus and chance-adjusted inter-rater agreements were calculated.

### The rating of overall reporting quality

We defined the overall quality of reporting as the degree to which the rationale, design, conduct and analysis of each RCT, evaluated in this study, were reported. In order to do this, 15 relevant items from the 2010 CONSORT statement were adopted in this quality of reporting evaluation of RCTs (Table 1). These 15 items were chosen because of literature indicating that an absence in their reporting is associated with a higher level of bias [6]. The CONSORT discussion items were excluded because of the subjective nature in assessing them. Three key methodological qualities that are part of the CONSORT statement were also included in a separate assessment. An Overall Quality of Reporting Score (OQRS), with 15 items in total (score out of 15), was established. Each of the 15 items was scored 1 if it was reported and 0 if it was not clearly stated or definitely not stated. Each RCT assessed could have a possible score between 0 and 15 inclusively. Two additional items that are considered important to be present in RCT publications were also assessed (Table 1), however they were not included in the calculation of the OQRS. This included a participant flow chart, as indicated by the CONSORT statement [6], and an ethical item, as it can improve a clinical study’s review of its methodology and patient safety [30].

**Table 1:** Description of overall quality of reporting items

**Item**	**Description**
1. Title or Abstract	Identification as a randomized trial in the title and a structured summary of the trial design, methods, results, and conclusions.
**Introduction**	
2. Background	Scientific background and explanation of rationale.
3. Objectives	Specific objectives or hypotheses.
**Methods**	
4. Participants	Eligibility criteria for participants. Settings and locations where the data were collected
5. Interventions	The interventions for each group with sufficient details to allow replication, including how and when they were actually administered.
6. Outcomes	Completely defined pre-specified primary and secondary outcome measures, including how and when they were assessed.
7. Sample Size	How sample size was determined. When applicable, explanation of any interim analyses and stopping guidelines.
8. Randomization: Sequence Generation	Method used to generate the random allocation sequence Type of randomisation; details of any restriction (such as blocking and block size).
9. Randomization: Implementation	There is mention of: Who generated the random allocation sequence, who enrolled participants, and who assigned participants to interventions?
10. Statistical Methods	Statistical methods used to compare groups for primary and secondary outcomes. Methods for additional analyses, such as subgroup analyses and adjusted analyses.
**Results**	
11. Participants Flow	For each group, the numbers of participants who were randomly assigned, received intended treatment, and were analysed for the primary outcome. For each group, losses and exclusions after randomisation, together with reasons.
12. Recruitment	Dates defining the periods of recruitment and follow-up. Why the trial ended or was stopped.
13. Baseline Data	A table showing baseline demographic and clinical characteristics for each group.
14. Outcomes and Estimates	For each primary and secondary outcome, results for each group, and the estimated effect size and its precision (such as 95% confidence interval). For binary outcomes, presentation of both absolute and relative effect sizes is recommended.
15. Harms	All important harms or unintended effects in each group
**Additional Items**	
16. Ethical Issues	The approval of an ethics committee and obtaining of informed consent from participants are stated
17. Flowchart	A flowchart of participants in each stage of the RCT (randomization, allocation, follow-up, and analysis for primary outcome) is provided

Please note that the description describing each CONSORT item used are taken directly from the “CONSORT 2010 Statement: updated guidelines for reporting parallel group randomised trials” article [1].

### Rating of key methodology items

Appropriate concealment of allocation mechanisms, blinding, and numbers analysed (intention-to-treat principle) were assessed separately because they are highly important in avoiding bias and distortions of the effect estimates [4,9,26,31-35]. The methodological items are also underreported in studies with a high overall reporting quality score hence emphasizing the need to assess them separately [34,35].

Appropriate concealment of allocation is a process that assigns patients in an RCT into alternative treatment groups in a way that will prevent the people who manage the allocation of patients as well as the patients themselves from foreknowledge of the allocation mechanism. This is done in order to avoid selection bias [8]. Obvious and easily manipulated variables such as medical record numbers or birthdays are not adequate for use in allocation concealment mechanisms. In order to avoid selection bias, allocation concealment mechanisms considered appropriate by the Cochrane Handbook are centralized randomization schemes, numbered, coded vehicles and sequentially numbered, sealed and opaque envelopes [5]. Hence, we considered allocation concealment to be appropriate in this study if centralized randomization, numbered, coded vehicles, or opaque, sealed, and sequentially numbered envelopes were reported [8].

Blinding refers to the process by which study participants, health care providers, outcome assessors, data assessors and manuscript writers are kept unaware of the intervention to which patients are randomized. Blinding helps to reduce the risk of knowing which intervention was received and this is critical, as knowledge of the assigned intervention can affect outcomes and assessments of outcomes [8,36]. Physicians and textbooks vary in their understanding and definitions of single, double, and triple blinding thus making the sole use of these terms in a study ambiguous in describing the blinding method. Despite this, single, double and triple blinding are terms commonly and singularly used to report blinding status without the accompaniment of descriptors indicating who was indeed blinded [36-38]. A meta-analysis indicated that double blinding is associated with potential bias reduction; double blinded studies reported a 15% lower treatment effect than non-blinded studies [4]. Sometimes there are cases where the blinding of patients and health care providers is not feasible. This is especially true in instances where there is a comparison of the effectiveness of a minimally invasive procedure with a procedure that is more invasive, as with surgery. In this case, patients or investigators cannot easily be blinded [38]. In these instances, where blinding is not feasible, we considered blinding to have been done appropriately if at least one specific group was unambiguously reported as blinded [3]. In RCTs where blinding did not have any barriers, at least two groups had to be unambiguously reported as blinded to fulfill this methodological item criteria [3,8,38].

We defined the numbers analyzed based on the intention-to-treat (ITT) principle. In this study we define ITT analysis to include all participants randomized into a trial and its analysis regardless if treatment or the intervention was administered, if patients satisfied the entry criteria, if patients withdrew from the study, or if there were deviations from the protocol [8,39-41]. Though the meaning of ITT has been interpreted differently by numerous RCTs, the Cochrane Handbook has compiled evidence indicating that the definition we have used prevents biased treatment effects and is a pragmatic way to understand the effects of a clinical intervention [41,42].

Each of the three above mentioned methodological items was scored 1 point if the method was appropriately reported and performed and 0 points if it was inappropriate or ambiguous. A combined methodological score of these three items was calculated for each trial by adding the scores of each item with a possible score ranging from 0 to 3 inclusively. This score was termed Key Methodological Item Score (KMIS). An inter-rater agreement was calculated for each of the three included methodological items as done in a previous study [3].

### Definition of predictor variables

Sample size was defined as the number of patients randomized in each trial. The journal impact factor refers to an index number assigned and calculated by Thomson Reuters to a particular journal catalogued in Thomson Reuter's Journal Citation Reports that indicates the frequency with which the "average article" in a specific journal has been cited in a particular year or period [43]. Funding reported was defined as funding mentioned and provided for the conduct of the study. Journal adoption of the CONSORT statement at the time of data abstraction was defined as the article’s journal endorsing, encouraging or enforcing the CONSORT statement for study submissions at the time the article was reviewed for this study.

### Hypotheses

Journal impact factor, sample size and declared funding have been correlated with better reporting [3,7,25,44-48]. Journal adoption of the CONSORT statement has also been associated with improved reporting of RCTs [6,7,10,11,22]. We hypothesized that studies with larger sample sizes, that have funding reported, that are published in journals with a higher impact factor and that are in journals that have adopted, endorsed or supported the CONSORT statement will be associated with better quality of reporting and reporting of key methodological items.

### Statistical analysis

The statistical analysis was modeled based on the analysis done in a previous study looking at the quality of reporting of general endocrinology literature [3]. The predictor variables, journal impact factor and sample size were also assessed in this study [3]. The new predictor variable included for our study is journal adoption, endorsement or support of the CONSORT statement at the time of data abstraction and funding reported.

Categorical data were reported as the number of counts. The number of trials that clearly stated the element and the associated 95% confidence interval (CI) were calculated. For the element that had “zero” count or “full” counts (i.e. when none of or all of the included trials reported that element respectively), the 95% CI was calculated by adopting the rule of three [49]. If none of the n studies showed the element that we were interested in, we could be 95% confident that the chance of this event occurring is at most 3 in n [49]. For the other elements, the 95% CI of the count was calculated by assuming that the number of trials that clearly stated the element followed a binomial distribution. The probability that a trial had clearly stated the element was set to be the observed probability in the sample. The Cohen’s Kappa (κ) statistics were used to calculate the chance-adjusted agreement between the two raters for the 15 quality of reporting items, the 2 additional items (ethical issues and flow chart) and the 3 key methodological items. Agreement was interpreted as poor if κ ≤ 0.2; fair if 0.21 ≤ κ ≤ 0.4; moderate if 0.41 ≤ κ ≤0.6; substantial if 0.61 ≤ κ ≤ 0.8; and good if κ > 0.8 [50].

A multivariable regression analysis with the OQRS as an outcome variable was conducted to determine if a higher sample size, a higher impact factor, the presence of funding reported and the journal adoption, endorsement or support of the CONSORT statement was independently associated with a better OQRS. These four variables were used as predictors. The sample size was transformed by the logarithm function with base 10 to improve interpretability. The OQRS (discrete, ranged from 0–15) was assumed to follow a Poisson distribution. Incidence rate ratios (IRR) were used to express the results of the analysis. Variables were considered to be statistically significant at alpha (α) = 0.05. The same approach was followed for regression analysis with the KMIS as the outcome variable. It is also important to note that we used Variance Inflation Factors (VIFs) to assess collinearity between predictors and none of the VIFs were greater than 10 showing no major collinearity among the variables. SPSS© 19.0.0.0 (IBM Corporation, 2010) was used to perform the statistical analysis.

## Results

### Rating of quality of reporting

The inter-rater agreement (κ) for the quality of reporting items varied from 0.646 to 1, indicating substantial to perfect agreement. The ratings of items for the OQRS are shown in Table 2. Some factors that constitute the overall quality of reporting score were consistently well reported. These were items 2, 3, 5, and 13 (Background, Objectives, Intervention and Baseline Data respectively) and they were properly reported in over 90% of the studies. The other items were poorly reported in the majority of the RCTs evaluated. Seven of the 15 items of the overall quality of reporting score were correctly reported in fewer than 50% of the studies. These items were Title and Abstract, Participants, Outcomes, Randomization: Sequence Generation, Randomization: Implementation, Recruitment, and Outcomes and Estimates. The mean OQRS was 8.43 (SD = 1.93), and the median was 9.0 (IQR = 3), with the minimum and maximum score of 4 and 11 respectively. Among the additional items considered, ethical issues were consistently well reported in 21 out of the 23 studies while a participant flow chart was provided in only 2 of the RCTs evaluated (Table 2).

**Table 2:** Overall Quality of Reporting: Rating using items from the CONSORT Statement and two additional items

**Item**	**All Articles (n = 23)**	
	**Count**	95% CI*
**1. Title or Abstract**	0	(0, 8)
**2. Background**	22	(20, 23)
**3. Objectives**	23	(15, 23)
**4. Participants**	6	(2, 10)
**5. Interventions**	23	(15, 23)
**6. Outcomes**	9	(5, 14)
**7. Sample Size**	19	(15, 22)
**8. Randomization: Sequence Generation**	8	(4, 13)
**9. Randomization: Implementation**	1	(0, 3)
**10. Statistical Methods**	17	(13, 21)
**11. Participants Flow**	12	(7, 17)
**12. Recruitment**	3	(0, 6)
**13. Baseline Data**	22	(20, 23)
**14. Outcomes and Estimates**	11	(6, 16)
**15. Harms**	18	(14, 22)
**a. Ethical Issues**	21	(18, 23)
**b. Flowchart**	2	(0, 5)

CI: Confidence Interval.

*For item that has non-zero event, the 95% CI was approximated by assuming the number of events followed a Binomial distribution; for item that has zero event, the 95% CI was approximated by the rule of three [49].

### Rating of key methodological items

The inter-rater agreement (κ) for the quality of key methodological items was 1 for all items, thus there was perfect agreement. The number of studies that reported each key methodological item is provided in Table 3. Among the 23 RCTs, 7 (30.4%) did not report any of the three key methodological items and none of the 23 studies evaluated reported all three methodological items. The reporting of the key methodological items among studies with an OQRS above the 75^th^ percentile was also poor. The average key methodological score of articles with an OQRS above the 75^th^ percentile was 1. Of the articles above the 75^th^ percentile for the overall quality of reporting score only 3 articles describe an appropriate allocation concealment mechanism, 1 article describes blinding and 1 article describes intention-to-treat analysis. Consequently, those studies scoring a higher OQRS did not guarantee a good report of key methodological items.

**Table 3:** Reporting Quality of Key Methodology Items

**Criteria**	**Description**	**All Articles (n = 23)**	
		**Count**	95% CI*
**Allocation of Concealment**	Allocation was considered appropriate in a RCT study if one of the following allocation methods were reported; 1) central randomization, 2) numbered, coded vehicles, and 3) opaque, sealed, and sequentially numbered envelopes	8	(4, 13)
**Blinding**	Blinding was considered to have occurred if at least one specific group was explicitly reported as blinded if there was a blinding feasibility issue. In cases where blinding was not an issue, at least two groups must have been explicitly reported as blinding to qualify as appropriate blinding.	5	(1, 9)
**Numbers Analysed (Intention to treat)**	Intention to treat was defined as the inclusion of all patients randomly assigned in the analysis, regardless of whether they actually satisfied the entry criteria, the treatment actually received, and subsequent with withdrawal or protocol deviations.	9	(5, 14)

CI: Confidence Interval.

*For item that has non-zero event, the 95% CI was approximated by assuming the number of events followed a Binomial distribution; for item that has zero event, the 95% CI was approximated by the rule of three [49].

An appropriate method of allocation concealment was reported in only 8 of the 23 RCTs (34.8%), with opaque, sealed and sequentially numbered envelopes being the only method reported by all of these 8 RCTs. Most reports, 15 of 23 (65.2%) did not give any information about this key methodological item.

Only 6 of the 23 RCTs (26.1%) were blinded according to the study definition. Among the 18 studies where blinding of patients was considered feasible, only 4 of those 18 studies specifically reported at least two blinded groups. Among the 5 studies where blinding of patients was considered not feasible, 2 reported blinding of another group. Table 3 shows the number of studies reporting key methodological items.

### Factors associated with reporting quality

Table 4 displays the results of the multivariable analysis of factors associated with the OQRS. Though the regression model did not show a significant association (*p* < 0.05) for any of these four predictor variables (sample size, impact factor, journal adoption of the CONSORT statement at the time of data collection and funding reported) the model did show a trend for funding reported, where the presence of funding reported in a study showed an observed 26.2% more quality of reporting items present or higher than studies without funding reported, on average.

**Table 4:** **Regression analysis for the Overall Reporting Quality**^**a**^**in 23 RCTs**

**Predictor variable**	**IRR**	**95% CI**	***P*value**
Sample Size^b^	0.791	(0.326, 1.919)	0.605
Impact Factor	0.934	(0.722, 1.208)	0.604
Journal adopted CONSORT Statement at the time of data collection.	0.938	(0.671, 1.312)	0.708
Funding reported	1.262	(0.837, 1.903)	0.266

CI: Confidence Interval; IRR: Incidence Rate Ratio.

^a^ Maximum Possible Score for the Overall Reporting Quality = 15.

^b^ The sample size variable was log(10) transformed. The value is an expression of the change in the average of the OQRS due to one unit increase in sample size in the log scale.

The multivariable analysis of factors associated with better reporting of KMIS also did not show a significant association for any of these four predictor variables (Table 5). Nonetheless the model did show a trend for funding reported as was shown with the regression for the overall quality score, where the presence of funding reported in a study showed an observation of 76.6% more key methodological items present or higher than studies without funding reported, on average. In addition, the model showed a trend for impact factor, where a 1 unit increment in impact factor showed an observation of 102.2% more key methodological items present.

**Table 5:** **Regression analysis for the Key Methodology Item Score**^**a**^**in 23 RCTs**

**Predictor variable**	**IRR**	**95% CI**	***P*value**
Sample Size^b^	2.383	(0.191, 29.665)	0.500
Impact Factor	2.022	(0.742, 5.509)	0.169
Journal adopted CONSORT Statement at the time of data collection.	0.517	(0.148, 1.803)	0.301
Funding reported	1.766	(0.619, 5.040)	0.288

CI: Confidence Interval; IRR: Incidence Rate Ratio.

^a^ Maximum Possible Score for the Key Methodology Item = 3.

^b^ The sample size variable was log(10) transformed. The value is an expression of the change in the average of the KMIS due to one unit increase in sample size in the log scale.

## Discussion

In this study we assessed the RCT reporting quality of 23 RCTs used within a FNB meta-analysis [29]. Most of these articles came from anesthesia specialty journals, except for 3 of the RCTs. All RCTs were related to post-operative management of analgesia after total knee arthroplasty. The overall quality of reporting of the RCTs assessed was poor to moderate, with only 4 of the 15 items being reported in over 90% of the articles. We have identified several areas where reporting of a particular item was insufficient in most of the studies assessed. These areas included: Item 1) Title and Abstract, Item 4) Participants, Item 6) Outcomes, Item 8) Randomization: Sequence Generation, Item 9) Randomization: Implementation, Item 11) Participants Flow, Item 12) Recruitment and Item 14) Outcomes and Estimation. Areas where items were reported moderately well were: Item 7) Sample size, Item 10) Statistical Methods and Item 15) Harms. Also very noteworthy was that the reporting of the key methodological items was poor with less than 50% of the articles reporting any of the 3 key methodological items (Table 3). Our results agree with many studies which have assessed the quality of reporting of RCTs published in medical and subspecialty journals, including studies which have focused on journals specialized in anesthesia [3,17,21,23-29,44-46]. All of these studies showed there is poor quality of reporting, with the 3 key methodological items assessed in this study (appropriate allocation concealment mechanism, blinding and numbers analysed by ITT) being highlighted as poorly reported [17,21,23-27,35,44]. Even though there is evidence to suggest that the quality of reporting has significantly improved over time, especially with the onset, use and publication of the CONSORT statement, the poor quality of reporting overall suggests that the statistically significant improvements are not enough to be clinically important as higher quality of reporting is needed to reduce bias and properly support clinicians’ decisions about treatment management [13,20,22,35]. Also, the overall quality of reporting does not necessarily correspond with the proper reporting of the 3 key methodological items assessed for in this study; 3 items which are deemed important in preventing bias [9,35]. Allocation concealment is important in avoiding selection bias, proper blinding is important in avoiding performance and detection bias, and numbers analyzed (ITT principle) is important in avoiding attrition bias [9]. Any method of randomization, allocation concealment or blinding which allows the investigator to control the treatment group to which the study participant will be assigned should be avoided [27]. Recent studies suggest that deficiency and poor quality of reporting of these 3 key methodological items often causes bias in the estimate of treatment effects, though the extent of bias can be unpredictable [27,31,32]. The overall poor quality of reporting in anesthesia literature is important to highlight as it suggests that there is a need to standardize the reporting of RCTs in anesthesia publications where information is retrieved, disseminated, and used for clinical practice. Although the CONSORT group was created to help improve reporting, there seems to be a need to register the urgency for completeness, clarity and transparency of reporting [1] in order to make publications more accessible and easily comparable with one another. This is apparent with the 23 RCTs assessed for this study as well since all of these articles were published after the CONSORT statement was established in 1996. The introduction of the CONSORT among journals and enforcing its use by requiring authors to submit a CONSORT checklist has the potential to greatly improve the quality of reporting for its readers and researchers needing to use the important information that RCTs provide for future application and meta-analysis. This notion is supported by evidence shown in 3 recent observational studies demonstrating that the introduction of the CONSORT statement is associated with an improvement in the quality of reporting [20,22,51].

### Limitations

There are many limitations to this study. One of the key limitations is the small sample size of RCTs used to evaluate the quality of reporting and key methodological items. The reason for this is that our intention was to evaluate the reporting quality of RCTs used in an actual meta-analysis. In hindsight, our study may not have had enough power to detect any significant association with our four predictor variables. In order to compensate for this we looked at trends among our findings with two other studies [3,25] that looked at analogous predictor variables using comparable methods of statistical analysis, and their association with the overall quality of reporting or reporting of key methodological items in RCTs. We found that two of the predictor variables we looked at (Sample Size and Impact Factor) had shown a significant association with reporting quality in these two studies. We were not able to compare our other two predictor variables (Journal adoption of CONSORT statement at the time of data collection and funding reported) with these two comparator studies and with other studies that were reviewed because of one of three reasons: 1) the variable was not used as a predictor variable, 2) the findings were not reported in a numerical and statistical manner that could be used for trend comparison, or 3) it was not reported at all in publication. For the OQRS there were a few noted trends. Though the direction and the magnitude of the effect of sample size was not seen as similar to the two comparator studies, our 95% CI range for sample size encompassed the IRR of both comparator studies suggesting that there is a possibility that our results would have showed association had the sample size been larger. However, for the predictor variable of impact factor, which was measured in our study on a continuous scale, there were no trends observed in the one study we could use as a comparison. It should be noted that the comparator paper we could use for impact factor measured impact factor association in a categorical manner. Most of the impact factors of the RCT journals in our study fell into only one of those categories suggesting that our CI range may have encompassed their model had we been able to compare on a continuous scale. We also had 4 RCTs that came from journals with no impact factor evaluation for 2010. This decreased our RCT sample size from 23 to 19, further reducing our power to detect a difference.

For the KMIS there were also a few noted trends with one of the comparator studies. Both the sample size and impact factor of our results matched the direction and range found in the study by Lai et al. suggesting that our results would have reached significance had the sample size been larger. This trend is also more likely to be apparent with KMIS, even with the sample size we had, because of noted studies suggesting that key methodological items, such as the three used in this study, are associated with improved quality of reporting [3,7,25,44-46] and the importance the 3 key methodological items assessed (Blinding, Allocation Concealment and Numbers Analysed) have in reducing bias [6,31,32,36,52]. In the future it might be of interest to assess the quality of RCTs from more than one meta-analysis on a related topic or even a random sample of RCTs in recent years in primary journals. Another limitation of our study is the fact that we cannot verify the degree with which the quality of reporting reflects the true methodological quality of the RCTs we assessed. The lack of reporting of a particular item in an RCT does not mean that the study in truth had a poor methodology. It is possible that the protocols of many RCTs include important aspects of methodology however important methodological details were left out of the published report [53,54]. In one study it was reported that the proportion of double blinded trials with a clear description of the blinding of participants increased from 19%, with reliance on the publication alone, to 67% when the protocol for the RCT was reviewed and supplemented for assessment with the publication [55]. Although the reporting on the key methodological item of blinding was found to be inadequate in both trial protocols and publications, the results show that there is still a possibility that the methodological and reporting quality of an RCT is greater than what is assessed in the published report [55,56]. Soares et al. also showed that adequate concealment of allocation was achieved in all protocols but was only reported in 42% of the published papers [56]. Reviewing protocols may improve the quality of reporting assessment however the published report is an important proxy in determining the validity and clinically relevant effect estimate as it is the source most accessible to clinicians and researchers. The quality of the report itself is important for this purpose. An additional limitation was the fact there is no standard instrument to evaluate the quality of reporting of RCTs. The majority of scales have not been thoroughly developed or tested for in regards to validity and reliability as a gold standard (criterion validity) is needed to compare it against. A gold standard does not exist for assessing the quality of RCTs and so checklists demarcating important features of quality and scales based on a quality of reporting checklist are assessed based on face validity and content validity from a theoretical model founded on accepted criteria [57,58]. Quality scores based on checklists are also unreliable and may introduce bias as scores were found to differ depending on the scoring system that was used [59,60]. Our results may also not entirely represent the quality of reporting of RCTs used in meta-analyses related to the intervention of the meta-analysis we used [29]. Therefore the assessment of the quality of reporting of the RCTs used in other anesthesia related meta-analyses may help in compiling a sufficient pool of studies providing a sample size with enough power to detect a significant difference. Even with our limitations our results have good internal validity as we had two qualified reviewers and good inter-rater correlation despite that there was a lack of clarity with the reporting of evaluated items. In the future, one important item to add to the OQRS is trial registration. Trial registration is another recently enforced part of any published RCT article in order to ensure accessibility of all research. Studies have shown that the presence of registration with a trial registry is suboptimal [61,62] and trial registration is associated with improved reporting in RCTs within the highest ranked journals [62]. It is also important to note that the year of publication definitely plays a role in the reporting quality of the RCTs chosen for our study. The CONSORT statement was established in 1996 and the speed of implementation of the guideline from the CONSORT group has been substandard as there continue to be observational studies concluding inadequate quality of reporting of many RCTs dated from 1996 till now [1,17,21,23-25]. Indeed, it has been shown that the total quality of reporting score is significantly associated with factors such as trial size, publication year, and the impact factor which the RCT is published within [44,47,48]. With that said the medical research community should also take notice that impact factor has been shown to be statistically significant as a predictor for better reporting quality on several occasions [25,47,48] and may be a feature to look for when wanting to find RCT publications that are of high reporting quality. It is possible that a large proportion of journals that have a higher impact factor have made initiatives to have authors submitting RCT manuscripts to their journal to adhere to the CONSORT statement. This may be worth investigating in the future to see if such a relationship exists.

## Conclusions

In conclusion our study showed that the quality of reporting and reporting of key methodological items within anesthesia RCTs used for a post-operative pain management meta-analysis is poor to moderate. The knowledge gained from this study should be taken as another opportunity for journals to register the urgency for RCT publications to be complete, clear and transparent in reporting in order to make the literature accessible and comparable. Enforcing the use of the CONSORT statement by requiring authors to submit a CONSORT checklist can help greatly to improve the quality of reporting and get authors in the frame of reference needed to properly report studies or even structure study design.

## Abbreviations

CI, Confidence Interval; CONSORT, Consolidated Standards for Reporting Trials; FNB, Femoral Nerve Block; IRR, Incidence Rate Ratio; IQR, Inter-Quartile Range; KMIS, Key Methodological Item Score; PCA, Patient-Controlled Analgesia; RCT, Randomized Controlled Trial; OQRS, Overall Quality of Reporting; SPSS, Statistical Package for the Social Sciences.

## Competing interests

The authors declare that they have no competing interests.

## Authors' contributions

VBD was involved with the conception of the study, participated in the study design, the acquisition of data, helped to perform the statistical analysis, made substantial contributions to the interpretation of the data, and drafted the manuscript. SZ was substantially involved with data acquisition and critical revisions to the paper. CY participated in the statistical design of the study, performed and made substantial contribution to the statistical analysis and interpretation of data and helped critically revise the manuscript. LT was involved with the conception of the study and made substantial contributions to the statistical analysis, interpretation of data and critically revised the manuscript. JP, LH, AA and YM were involved with contributing data and helped draft and edit the final manuscript. All authors read and approved the final manuscript.

## Pre-publication history

The pre-publication history for this paper can be accessed here:

http://www.biomedcentral.com/1471-2253/12/13/prepub
